# Allele-specific silencing as therapy for familial amyotrophic lateral sclerosis caused by the p.G376D *TARDBP* mutation

**DOI:** 10.1093/braincomms/fcac315

**Published:** 2022-12-16

**Authors:** Roberta Romano, Maria De Luca, Victoria Stefania Del Fiore, Martina Pecoraro, Serena Lattante, Mario Sabatelli, Vincenzo La Bella, Cecilia Bucci

**Affiliations:** Department of Biological and Environmental Sciences and Technologies (DiSTeBA), Via Provinciale Lecce-Monteroni n.165, 73100 Lecce, Italy; Department of Biological and Environmental Sciences and Technologies (DiSTeBA), Via Provinciale Lecce-Monteroni n.165, 73100 Lecce, Italy; Department of Biological and Environmental Sciences and Technologies (DiSTeBA), Via Provinciale Lecce-Monteroni n.165, 73100 Lecce, Italy; ALS Clinical Research Center, P Giaccone University Hospital and Department of Biomedicine, Neuroscience and advanced Diagnostic (BIND), University of Palermo, via Gaetano La Loggia n° 1, 90129 Palermo, Italy; Section of Genomic Medicine, Department of Life Sciences and Public Health, Università Cattolica del Sacro Cuore, Rome, Italy; Unit of Medical Genetics, Department of Laboratory and Infectious Disease Sciences, Fondazione Policlinico Universitario A. Gemelli IRCCS, Rome, Italy; Adult NEMO Clinical Center, Unit of Neurology, Department of Aging, Neurological, Orthopedic and Head-Neck Sciences, Fondazione Policlinico Universitario A. Gemelli IRCCS, Rome, Italy; Section of Neurology, Department of Neuroscience, Faculty of Medicine and Surgery, Università Cattolica del Sacro Cuore, Rome, Italy; ALS Clinical Research Center, P Giaccone University Hospital and Department of Biomedicine, Neuroscience and advanced Diagnostic (BIND), University of Palermo, via Gaetano La Loggia n° 1, 90129 Palermo, Italy; Department of Biological and Environmental Sciences and Technologies (DiSTeBA), Via Provinciale Lecce-Monteroni n.165, 73100 Lecce, Italy

**Keywords:** ALS, TDP-43, siRNA therapy, antisense oligonucleotides, allele-specific silencing

## Abstract

Amyotrophic lateral sclerosis is a neurodegenerative disease characterized by the degeneration of motor neurons. There is no treatment for this disease that affects the ability to move, eat, speak and finally breathe, causing death. In an Italian family, a heterozygous pathogenic missense variant has been previously discovered in Exon 6 of the gene *TARDBP* encoding the TAR DNA-binding protein 43 protein. Here, we developed a potential therapeutic tool based on allele-specific small interfering RNAs for familial amyotrophic lateral sclerosis with the heterozygous missense mutation c.1127G>A. We designed a small interfering RNA that was able to diminish specifically the expression of the exogenous Green Fluorescent Protein (TAR DNA-binding protein 43^G376D^ mutant protein) in HEK-293T cells but not that of the Green Fluorescent Protein (TAR DNA-binding protein 43 wild-type). Similarly, this small interfering RNA silenced the mutated allele in fibroblasts derived from patients with amyotrophic lateral sclerosis but did not silence the wild-type gene in control fibroblasts. In addition, we established that silencing the mutated allele was able to strongly reduce the pathological cellular phenotypes induced by TAR DNA-binding protein 43^G376D^ expression, such as the presence of cytoplasmic aggregates. Thus, we have identified a small interfering RNA that could be used to silence specifically the mutated allele to try a targeted therapy for patients carrying the p.G376D TAR DNA-binding protein 43 mutation.

## Teaser

Romano *et al*. identified a small interfering RNA able to specifically silence the allele of the *TARDBP* gene mutated in a familial form of amyotrophic lateral sclerosis due to the heterozygous missense mutation c.1127G>A. The treatment with this siRNA reverted the pathological phenotype in cellular models, representing a potential therapeutic tool.

## Graphical Abstract

**Graphical abstract:**
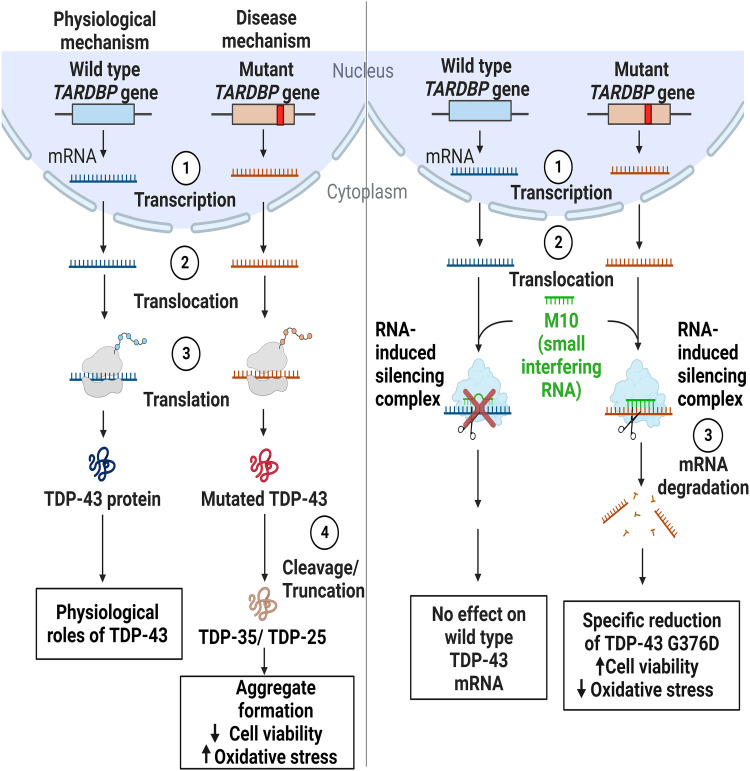

## Introduction

Amyotrophic lateral sclerosis (ALS) is an adult-onset neurodegenerative disease characterized by the loss of motor neurons caused by axonal degeneration and thus, the destruction of nerve terminals. The progressive loss of motor neurons leads to muscle weakness and wasting, affecting gradually the ability to speak, move, eat and eventually breathe, causing death.^1^ TAR DNA-binding protein 43 (TDP-43) is a major disease-related protein in sporadic and familial ALS.^2^ TDP-43 is a highly conserved, ubiquitously expressed nuclear protein involved in several steps of RNA metabolism, such as transcription, translation, mRNA transport, mRNA stabilization, microRNA and long non-coding RNA processing.^3^ TDP-43 is mainly in the nucleus, but it can shuttle between the nucleus and cytoplasm through active or passive transport.^4^ Regarding its structure, an N-terminal domain, two RNA recognition motifs (RRM1 and RRM2), a nuclear export signal and a C-terminal domain composed of TDP-43 protein.^2^ In the N-terminal domain is localized the nuclear localization sequence, mutations in which cause cytoplasmic aggregation of TDP-43.^5^ This protein plays an important role in transcriptional repression, pre-mRNA alternative splicing and translational regulation by binding UG/TG-rich single-stranded or double-stranded DNA/RNA through RRM domains.^6,7^ The C-terminal domain is rich in glycine, glutamine and asparagine, representing the prion-like domain and it is important for the solubility and cellular localization of TDP-43.^8,9^

Most ALS-related mutations are localized in the C-terminal domain.^10,11^ Indeed, it was demonstrated that G294V, A315T, M337V, A382T and G376D mutations, all lying in the C-terminal region, promote cytoplasmic mislocalization and aggregation. However, the mechanisms that underlie this phenotype are not yet known.^2^ ALS aggregates are characterized by the presence of truncated forms of TDP-43. In particular, the most frequent forms are the C-terminal fragments CTF35 (35 kDa) and CTF25 (25 kDa), which can be produced after Caspase 3, 4, and 7 cleavages.^12^ Therefore, the pathological hallmarks of TDP-43 include nucleus-to-cytoplasm mislocalization, protein truncation leading to the formation of toxic C-terminal TDP-43 fragments and protein aggregation and the deposition of ubiquitinated and hyperphosphorylated forms of TDP-43.^3,13–15^ At present, there is no treatment available that can prevent or reverse the neurodegenerative condition. Thus, downregulation of the mutant allele without suppression of the corresponding wild-type allele could be a promising therapeutic strategy. One way to target the mutant allele in cases of ALS is by using allele-specific small interfering RNAs (siRNAs), an approach that has been already described in several autosomal dominant diseases such as Parkinson’s disease,^16^ Alzheimer’s disease^17^ and Huntington’s disease.^18,19^ Silencing of a superoxide dismutase 1 (SOD1) mutant allele has been successfully achieved using siRNAs and short hairpin RNA in cells and animal models of ALS.^20–22^

A heterozygous missense mutation, 1127G>A in the Exon 6 of the *TARDBP* gene has been discovered in an Italian family with several members affected by ALS.^23^ To try to put the bases for treatment, we identified a siRNA complementary to the region comprising the point mutation c.1127G>A that specifically targets the TDP-43 mutant allele and that can counteract the cellular phenotypes induced by mutant allele expression.

## Materials and methods

### Antibodies

Primary antibodies used in this study were: rabbit polyclonal anti-TDP-43 (1:3000 for Western blot analysis, 10782-2-AP) and rabbit polyclonal anti-TDP-43 C-terminal (1:100 for immunofluorescence analysis, 12892-1-AP), both from Proteintech (Rosemont, IL, USA); rabbit polyclonal anti-histone H3 (1:10 000, ab1791) from Abcam (Cambridge, UK); rabbit polyclonal anti-glyceraldehyde-3-phosphate dehydrogenase (GAPDH) (1:2000, sc-25778) from Santa Cruz Biotechnology (Dallas, TX, USA).

### Cells and reagents

We used dermal fibroblasts derived from two healthy individuals (control fibroblasts) and two ALS patients belonging to an Apulian family from Lecce carrying the p.G376D TDP-43 mutation. Fibroblasts were previously obtained, after informed consent, by skin biopsies and then cultured under protocols approved by the Università Cattolica del Sacro Cuore Ethics Committee (Protocol P/740/CE/2012) and the Palermo 1 Ethics Committee (Protocols 7/2017 and 4/2019). HEK-293T and Neuro2A cells were from the ATCC. Fibroblasts were cultured in Dulbecco’s modified Eagle’s medium (DMEM) supplemented with 15% foetal bovine serum (FBS), 2 mM L-glutamine, 100 U/ml penicillin and 10 mg/ml streptomycin, while HEK-293T and Neuro2a cells were cultured in DMEM supplemented with 10% FBS, 2 mM L-glutamine, 100 U/ml penicillin and 10 mg/ml streptomycin in a 5% CO_2_ incubator at 37°C. We periodically checked that the cell lines used were free from mycoplasma infections. The reagents for tissue culture were from Sigma-Aldrich (St. Louis, MO, USA) or Gibco (Grand Island, NY, USA).

### Plasmid construction

The TDP-43^wt^ full-length construct used in this study has been described previously.^22^ We used the green fluorescent protein (GFP)-TDP-43^wt^ plasmid as a template to generate the GFP-TDP-43^G376D^ construct through the PfuTurbo DNA polymerase (Agilent Technologies, Santa Clara, CA, USA) following the manufacturer’s instructions and primers containing the desired mutation. The oligonucleotides used to generate the TDP43 mutant cDNA were 5′-GGAAATAACTCTTATATAGTGACTCTAATTCTGGTGCAGC-3′ and 5′-GCTGCACCAGAATTAGAGTCACTATAAGAGTTATTTCC-3′.

### Transfection and silencing

HEK-293T cells were transfected and silenced using oligofectamine transfection reagent (Invitrogen, Carlsbad, CA, USA) according to the manufacturer’s instruction and processed after 72 h, as previously described.^24^ Fibroblasts were silenced using Metafectene SI (Biontex, Martinsried, Germany) or Amaxa Cell Line Nucleofector Kit V (Lonza, Basel, Switzerland) according to the protocols provided by the manufacturers and then analysed after 6 days or 48 h, respectively. Neuro2A cells were transfected using Amaxa Cell Line Nucleofector Kit V, as previously described,^25^ or with Metafectene SI (Biontex, München, Germany) and processed 72 h after transfection. We designed siRNAs complementary to the region comprising the point mutation c.1127G>A in the mRNA of the human *TARDBP* gene. TDP-43 siRNAs are listed in Table 1. The siRNAs were produced by Eurofins Genomics (Ebersberg, Germany).

**Table 1:** siRNAs sequences

	Sequences
siM9	5′-UUAUAGUGACUCUAAUUCUdTdT-3′ 3′-dTdT AAUAUCACUGAGAUUAAGA-5′
siM10	5′-CUUAUAGUGACUCUAAUUCdTdT-3′ 3′-dTdTGAAUAUCACUGAGAUUAAG-5′
siM9/10	5′-UUAUAGUGAGUCUAAUUCUdTdT-3′ 3′-dTdT AAUAUCACUCAGAUUAAGA-5′
siM10/9	5′-CUUAUAGUCACUCUAAUUC-dTdxT3′ 3′-dTdTGAAUAUCAGUGAGAUUAAG-5′
TDP-43i	5′-AAGCAAAGCCAAGAUGAGCCU-dTdT-3′ 3′-dTdTAGGCUCAUCUUGGCUUUGCUU-5′

### Western blotting

HEK-293T cells and fibroblasts were lysed in Laemmli buffer [100 mM Tris–HCl, pH 6.8, 4% (w/v) sodium dodecyl sulphate (SDS), 0.2% (w/v) bromophenol blue, 20% glycerol and 200 mM dithiothreitol (dithiothreitol)] and subjected to Western blot analysis as previously described.^25–27^ Signals were visualized using Bio-Rad ChemiDoc MP Imaging Systems (Bio-Rad, Hercules, CA, USA) and densitometric analysis of band intensities was performed with Image Lab software (Bio-Rad).

### Confocal immunofluorescence

Fibroblasts and Neuro2A cells were treated as previously described.^28^ Briefly, cells were fixed with paraformaldehyde at 4% for 30 min at 37°C and then permeabilized using a solution of 10% Triton X-100 diluted 1:5000 in PBS. After 1 h of blocking, cells were incubated with the primary antibody for 40 min at room temperature and, after washes, with the secondary antibody for 20 min at room temperature. Coverslips were mounted using Mowiol solution. Images were acquired using Zeiss LSM700 confocal laser scanning microscopy (Zeiss, Oberkochen, Germany) or the EVOS FL Auto Cell Imaging System (Thermo Fisher Scientific, Waltham, MA, USA) and cells with aggregates were manually counted.

### Quantitative real-time polymerase chain reaction

RNA from HEK-293T and fibroblasts was extracted using the RNeasy Micro Kit according to the manufacturer’s instructions (Qiagen, Hilden, Germany). cDNA was prepared using SuperScript IV reverse transcriptase (Invitrogen) according to the manufacturer’s protocol. The polymerase chain reaction (PCR) reaction was prepared with SYBR™ Green PCR Master Mix (Applied Biosystem, Watham, MA, USA) and run in C1000 Touch Thermal Cycler (Bio-Rad), using the following thermal protocol: 94°C 5 min, 40 cycles of 94°C 30 s, 60°C 30 s, 72°C. The expression level was calculated with the 2^−ΔCT^ method as previously described.^26^ The normalization was performed using Rplp0 (ribosomal protein large P0).

The primers used were:

Rplp0: *forward*: 5′-TCGACAATGGCAGCATCTAC-3′
*reverse*: 5′-ATCCGTCTCCACAGACAAGG-3′
GFP: *forward*: 5′-CTGCTGCCCGACAACCAC-3′
*reverse*: 5′-TCACGAACTCCAGCAGGAC-3′
TDP-43: *forward*: 5′-GTATGATGGGCATGTTAGC-3′
*wild-type reverse:* 5′-CTGCACCAGAATTAGAGC-3′
*G376D reverse*: 5′-CTGCACCAGAATTAGAGT-3′

### Nuclear and cytoplasmic fractionation

Cells were lysed in lysis buffer (50 mM Tris pH 7.5, 0.1% Triton X-100, 137.5 mM NaCl, 10% glycerol, 5 mM EDTA) supplemented with Complete Protease Inhibitor Cocktail (Roche, Basel, Switzerland), and then incubated on ice for 15 min. After centrifugation at 7000 rpm for 2 min at 4°C, the supernatant, representing the cytosolic fraction, was collected. The pellet, which contained nuclei, was washed three times with lysis buffer. After the last centrifugation, the pellet containing the nuclear fraction was re-suspended in lysis buffer supplemented with 0.5% SDS. The fractions were then analysed by Western blot analysis.

### Cell viability assay

The sulforhodamine B (SRB) assay was performed to determine cell viability. Briefly, Neuro2A cells were seeded at the density of 2 × 10^4^ in a 24-well plate. After 24 h, cells were transfected with plasmids coding for GFP, GFP-TDP-43^wt^ and GFP-TDP-43^G376D^ and treated with control RNA or M10. After 72 h the medium was changed, and the cells were fixed with 125 µL of 50% cold (trichloroacetic acid) for 1 h at 4°C. The samples were washed five times with water and then let dry overnight at room temperature. 100 µL of SRB solution (0.4% SRB in 1% acetic acid) were added per well. After 30 min, the SRB solution was removed, and the cells were washed four times with 200 µL of 1% acetic acid. The retained SRB was solubilized by adding 800 µL of Tris 10 mM pH 10.5 per well for 10 min. Absorbance was read in a microplate reader (Victor X5, Perkin Elmer) at 565 nm.

### 2′,7′-Dichlorodihydrofluorescein diacetate staining

2′,7′-Dichlorodihydrofluorescein diacetate (DCFH-DA) staining was performed to determine total cellular reactive oxygen species (ROS) that underlies oxidative stress. Fibroblasts were seeded (2 × 10^4^ per well) in a 24-well plate, treated with control RNA or M10 for six days and then total ROS were detected by the DCFH-DA (Thermo Fisher Scientific) assay. The drug was added to each well at a concentration of 10 µM and incubated at 37°C for 30 min. Then, cells were washed three times with PBS, lysed in 100 µL of RIPA (radioimmunoprecipitation assay) buffer for 5 min on ice and centrifuged at 10 000 rpm for 10 min at 4°C. 90 µL of the supernatant were transferred to a 96-well plate and the fluorescence was read at 490 nm in a microplate reader (Victor X5, Perkin Elmer). The remaining part was used to measure protein concentration by Bradford assay and normalize fluorescence intensities with the protein concentration of each sample.

### Statistical analysis

The experiments were performed at least in triplicate. Data were statistically analysed using one-way ANOVA followed by Dunnett’s, Sidak’s or Tukey’s multiple comparison tests (*P < 0.05, **P < 0.01 and ***P < 0.001). Graphs represent mean value ± standard error mean (SEM).

## Results

### Pedigree of the family carrying the p.G376D TDP-43 mutation

**Fig. 1:**
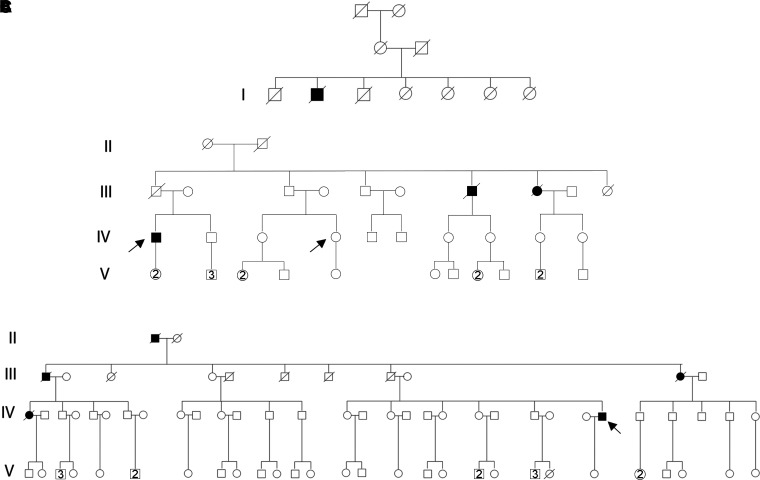
**Pedigree of the family affected by ALS for the TDP-43G376D mutation.** Square indicates male; circle female. Arrows indicate patients from which fibroblasts were obtained. Circles and squares crossed by a line indicate deceased female and male, respectively. (**A**) Family tree showing the first generation in which the first case of ALS was reported. (**B**) Family tree of Patient 1. (**C**) Family tree of Patient 2.

In this study, we performed our experiments using dermal fibroblasts from patients belonging to the same Apulian family from Lecce carrying the p.G376D TDP-43 mutation. The first case of ALS was reported in a man in the first generation of the family tree (Fig. 1A). In the second generation of this pedigree, there are seven branches, in three of which several members affected appear, for a total of 31 cases of ALS in this family, with 19 men and 12 women affected.

In our experiments, we used ALS1 and ALS2 cells: which were obtained from affected individuals belonging to the fourth generation of different branches of the family tree. We obtained dermal fibroblasts from an ALS1 patient when the disease was diagnosed (ALS1O, onset) and four years after diagnosis (ALS1A, advanced; IV.1, Fig. 1B). We also obtained dermal fibroblasts from a healthy woman of the same generation (CTRL2, IV.4, Fig. 1B) not carrying the p.G376D TDP43 mutation. ALS2A patient is indicated in Fig. 1C (IV.26). Clinical characteristics of patients are reported in Table 2.

**Table 2:** Clinical and demographic characteristics of the two TARDBP G376D ALS patients in the advanced stage included in the study

	ALS1A	ALS2A
Age of onset (years)	37	27
Disease duration from onset to tracheotomy (months)	16	10
Gender	Male	Male
Education (years)	13	8
ALSFRS-R at diagnosis (normal value: 48)	40	34
Disease progression (ΔFS) at diagnosis	1.33	1.56
Site of onset	Spinal	Spinal

ALSFRS-R= ALS functional rating scale revised^29^; ΔFS= rate of disease progression calculated as previously described.^30^.

### Identification of allele-specific siRNA for TDP-43^G376D^

We designed four allele-specific siRNAs targeting the c.1127G>A mutation that defined M9, M10, M9/10 and M10/9 (Table 1). Early studies on the RNAi pathway showed that a mismatch that occurs at the centre of the siRNA structure abolished the RNAi effect.^31^ Therefore, the position of the mutated nucleotide in the siRNA is important to discriminate the mutant from the wild-type allele. Allele-specific siRNAs targeting different regions of the same transcript display differences in silencing efficiency; therefore, we designed siRNAs, where the mutated nucleotide varies within the siRNA structure. The allele-specific siRNAs M9 (the mutated nucleotide is at Position 9) and M10 (the mutated nucleotide is at Position 10) are perfectly matched with the mutant allele but have a single mismatch with the wild-type counterpart. However, it is likely that not all single mismatches would create silencing selectivity, and some mismatches might be tolerated by the RNA-induced silencing complex, compromising the allele specificity.^32^ For this reason, we designed siRNAs containing a double mismatch with the wild-type (siM9/10 and siM10/9), hence creating a single mismatch with the mutant allele.

**Fig. 2:**
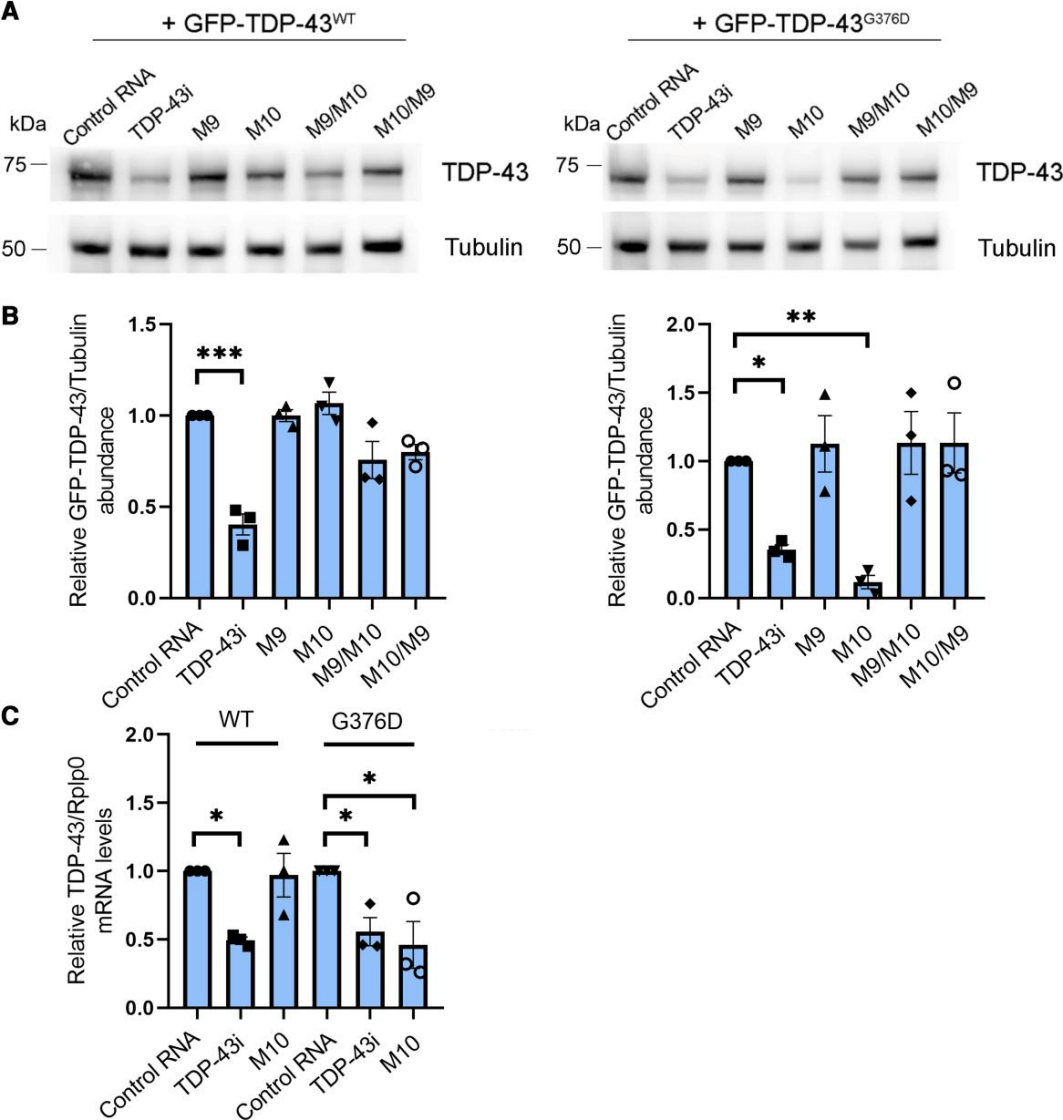
**An allele-specific siRNA downregulates the GFP-tagged TDP-43^G376D^ mutant allele.** (**A**) HEK-293T cells were transfected with plasmids encoding the GFP-TDP-43^WT^ or GFP-TDP-43^G376D^ proteins and with control RNA, TDP-43 siRNA or putative allele-specific siRNAs (M9, M10, M9/10 and M10/9). Cells were then lysed and analysed by Western blotting using specific anti-TDP-43 and anti-tubulin antibodies, in order to analyse GFP-TDP-43 levels and equal loading, respectively. See Supplementary Fig. 1 for uncropped blots. (**B**) Quantification of the amount of GFP-TDP-43 wild-type and mutant proteins upon transfection of the different siRNAs. Data are the mean ± SEM of at least three independent experiments. Statistical analysis was performed using one-way ANOVA followed by Dunnett’s multiple comparisons test. *P < 0.05; ** < 0.01; ***P < 0.001; *F* = 17.92 (for GFP-TDP-43^WT^); *F* = 8.488 (for GFP-TDP-43^G376D^). (**C**) The amount of GFP-TDP-43 transcript was quantified, compared with the Rplp0 transcript as control, using real-time PCR in HEK-293T cells transfected with GFP-TDP-43^WT^ or GFP-TDP-43^G376D^ plasmids and treated with control RNA, TDP-43 siRNA or allele-specific siRNA M10. Data represent the mean ± SEM of at least three experiments. Statistical analysis was performed using one-way ANOVA followed by Sidak’s multiple comparisons test. *P < 0.05; ** *P* < 0.01; ***P < 0.001; *F* = 6.606.

To determine if these allele-specific siRNAs specifically knock down the G376D allele, HEK-293T cells were transiently transfected with full-length GFP-TDP-43^wt^ or GFP-TDP-43^G376D^ in combination with allele-specific siRNAs for 72 h and analysed by Western blot. Non-targeting siRNA control (control RNA) and siRNA targeting the 5′ regions of the TDP-43 transcript (TDP-43i) were used as controls. As expected, TDP-43i was able to silence both GFP-TDP-43 wild-type and GFP-TDP-43^G376D^ (*P* = 0.0001 and *P* = 0.0477), while siM10 caused a significant reduction only of GFP-TDP-43^G376D^ protein levels (*P* = 0.0072), as GFP-TDP-43 wild-type levels remained unchanged by transfection (Fig. 2A and B).

Having determined that an allele-specific siRNA can reduce the protein abundance of the mutant allele specifically, we sought to determine whether this RNAi approach could induce a block of the translation or degradation of TDP-43^G376D^ mRNA; therefore, the expression of the corresponding mRNA was quantified by real-time PCR using primers against GFP. The analysis showed a substantial reduction of GFP-TDP-43 wild-type and GFP-TDP-43^G376D^ transcripts in the presence of TDP-43i (*P* = 0.0199 and *P* = 0.0436), while GFP-TDP-43^G376D^ transcript was reduced in the presence of siM10 (*P* = 0.0131), which did not influence the wild-type transcript abundance (Fig. 2C).

### Validation of allele-specific siRNA in fibroblasts isolated from ALS patients carrying the p.TDP-43 mutation

**Fig. 3:**
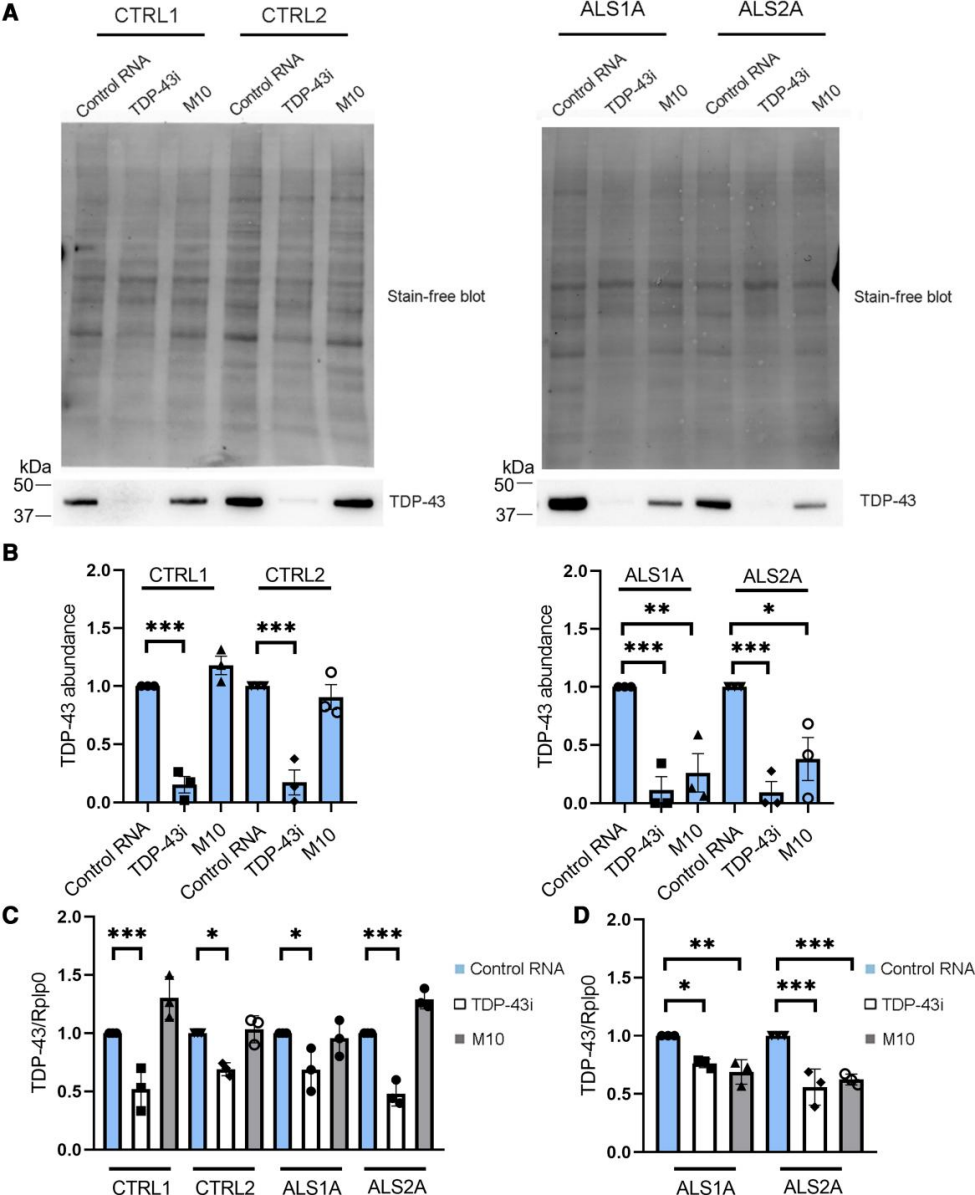
**Allele-specific siM10 decreases TDP-43^G376D^ abundance in ALS fibroblasts.** (**A**) Fibroblasts isolated from two controls and two ALS patients carrying the TDP-43^G376D^ mutation (ALS1, ALS2) were transfected with siM10, TDP-43i and control RNA for 6 days, lysed and subjected to Western blot analysis using anti-TDP-43 antibody and Stain-Free Imaging Technology (Bio-Rad) for loading control. See Supplementary Fig. 2 for uncropped blots. (**B**) Quantification of the amount of TDP-43 upon transfection with control RNA, TDP43 siRNA and allele-specific siRNA M10. Statistical analysis was performed using one-way ANOVA followed by Sidak’s multiple comparisons test. Data represent the mean ± SEM of at least three experiments. *P < 0.05; ** < 0.01; ***P < 0.001; *F* = 35.65 (for control cells); *F* = 12.77 (for ALS cells). (**C**) The amount of TDP-43 wild-type transcript was quantified, compared with the Rplp0 transcript as control, using real-time PCR in control and ALS fibroblasts treated with control RNA, TDP-43 siRNA or allele-specific siRNA M10. Data represent the mean ± SEM of at least three experiments. Statistical analysis was performed using one-way ANOVA followed by Sidak’s multiple comparisons test. *P < 0.05; ** < 0.01; ***P < 0.001; *F* = 16.03. (**D**) The amount of TDP-43 mutant transcript was quantified, compared with the Rplp0 transcript as control, using real-time PCR in ALS fibroblasts treated with control RNA, TDP-43 siRNA or allele-specific siRNA M10. Data represent the mean ± SEM of at least three experiments. Statistical analysis was performed using one-way ANOVA followed by Sidak’s multiple comparisons test. *P < 0.05; ** < 0.01; ***P < 0.001; *F* = 16.68.

In HEK-293T cells overexpressing GFP-TDP-43^G376D^, we showed that siM10 could selectively reduce the TDP-43^G376D^ protein amounts. To test whether allele-specific siRNA could decrease endogenous TDP-43^G376D^ levels in cells derived from patients, we transfected dermal fibroblasts previously isolated from ALS patients carrying the p.G376D TDP-43 mutation (ALS fibroblasts), and healthy individuals without the mutation (CTRL = control patients) with siM10, TDP-43i and control RNA. Western blot analysis showed that TDP-43i induced a significant reduction of TDP-43 abundance in control cells (CTRL1 *P* < 0.0001; CTRL2 *P* < 0.0001) and ALS fibroblasts (ALS1A *P* = 0.0007; ALS2A *P* = 0.0006), while transfection with allele-specific siM10 did not change the protein amount in controls but significantly reduced TDP-43 levels in ALS patient cells (ALS1A *P* = 0.0032; ALS2A *P* = 0.0114; Fig. 3A and B). Moreover, we performed a real-time PCR using a primer pair able to recognize the wild-type sequence of TDP-43. The transfection with siM10 did not change the TDP-43 wild-type mRNA in controls and patients while the treatment with TDP-43i significantly reduced TDP-43 transcript in controls (CTRL1 *P* = 0.0003; CTRL2 *P* = 0.0248) and patients (ALS1A *P* = 0.0216; ALS2A *P* < 0.0001; Fig. 3C). On the contrary, when we used a primer pair that specifically recognizes the mutant sequence, we observed reduced levels of TDP-43 transcript in ALS fibroblasts treated with TDP-43i (ALS1A *P* = 0.0138; ALS2A *P* < 0.0001), but mostly, a significant reduction of TDP-43^G376D^ mRNA levels after siM10 transfection (ALS1A *P* = 0.0018; ALS2A *P* = 0.0004; Fig. 3D).

### Allele-specific siRNA reduced cytoplasmic inclusion in ALS fibroblast carrying the p.G376D TDP-43 mutation and in Neuro2A cells transfected with the mutant plasmid

**Fig. 4:**
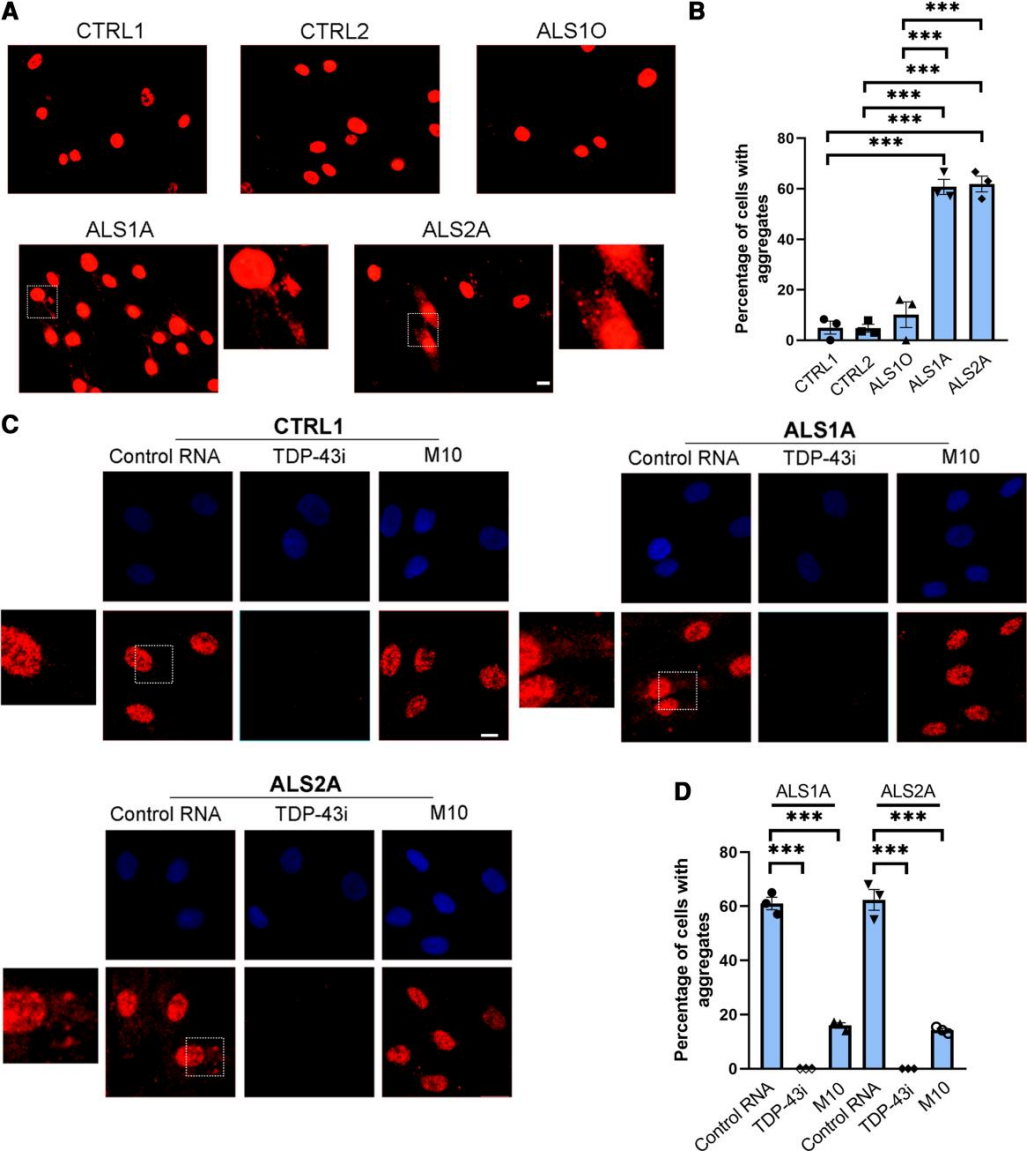
**Allele-specific siM10 decreases TDP-43 cytoplasmic inclusions in ALS fibroblasts.** (**A**) Control and ALS fibroblasts were fixed and immunolabelled with anti-C-terminal TDP-43 antibody followed by Alexa-568-conjugated secondary antibody. Bar 50 µM. (**B**) Quantification of the percentage of cells showing TDP-43 cytoplasmic aggregates. At least 50 cells per sample were analysed. Data represent the mean ± SEM of at least three experiments. Statistical analysis was performed using one-way ANOVA followed by Tukey’s multiple comparisons test. *P < 0.05; ** < 0.01; ***P < 0.001; *F* = 84.83. (**C**) Control and ALS fibroblasts treated with control RNA, TDP-43 siRNA or allele-specific siRNA M10 were fixed and immunolabelled with anti-C-terminal TDP-43 antibody followed by Alexa-568-conjugated secondary antibody. Nuclei were stained with DAPI. Bar 10 µM. Quantification of the percentage of cells with TDP-43 aggregates is also shown. At least 50 cells per sample were analysed. Data represent the mean ± SEM of at least three experiments. Statistical analysis was performed using one-way ANOVA followed by Dunnett’s multiple comparisons test. *P < 0.05; ** < 0.01; ***P < 0.001; *F* = 474.4 (for ALS1A); *F* = 209.6 (for ALS2A).

Both familial and sporadic ALS are characterized by TDP-43 cytoplasmic aggregates, which represent a hallmark of the disease.^12^ We used an antibody able to specifically recognize the C-terminal region of TDP-43 to visualize TDP-43 aggregates in fibroblasts by immunofluorescence experiments. We confirmed that cytoplasmic inclusions are visible in ALS1A and ALS2A patients but not in control cells. Moreover, we highlighted differences in ALS1 patient considering that TDP-43 aggregates are considerably more abundant in the stage of the advanced disease (ALS1A cells) compared with the early stage (ALS1O cells) (Fig. 4A). Indeed, the quantification of TDP-43 inclusions showed that about 60% of the cells of both patients present the aggregates while in ALS1O cells inclusions were not significantly different from control cells (Fig. 4B; *P* < 0.0001).

Having determined that ALS cells showed TDP-43 cytoplasmic aggregates, we wondered if allele-specific siRNA could reverse this phenotype. Confocal microscopy showed that M10 had no effect on control cells while it could strongly reduce the aggregates in both ALS fibroblasts. The quantification showed that the percentage of ALS1A and ALS2A fibroblasts with aggregates drops from about 60 to 15% following M10 treatment (ALS1A: control RNA/TDP-43i *P* < 0.0001; control RNA/M10 *P* < 0.0001; ALS2A: control RNA/TDP-43i *P* < 0.0001; cRNA/M10 *P* < 0.0001; Fig. 4C).

**Fig. 5:**
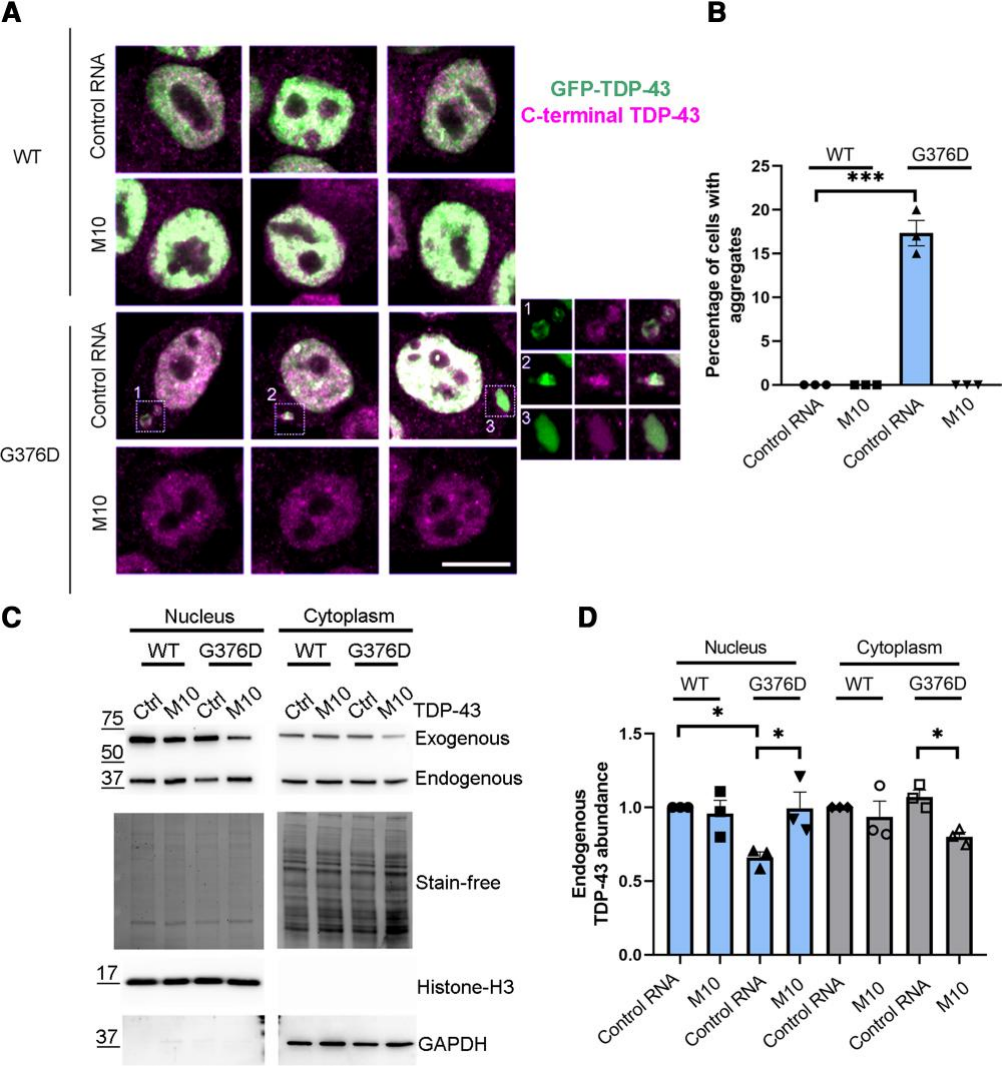
**Neuro2A cells transfected with GFP-TDP-43^G376D^ show aggregates which are no longer visible following siM10 treatment.** (**A**) Neuro2A cells were transfected with plasmids encoding the GFP-TDP-43^WT^ or GFP-TDP-43^G376D^ proteins and with control RNA or M10 siRNA. Seventy-two hours after transfection, cells were fixed and immunolabelled with anti-C-terminal TDP-43 antibody followed by Alexa-568-conjugated secondary antibody. Bar 10 µM. (**B**) Quantification of the percentage of cells showing TDP-43 cytoplasmic aggregates. At least 50 cells per sample were analysed. Data represent the mean ± SEM of at least three experiments. Statistical analysis was performed using one-way ANOVA followed by Dunnett’s multiple comparisons test. *P < 0.05; ** < 0.01; ***P < 0.001; *F* = 142.3. (**C**) HEK-293T cells were transfected with plasmids encoding the GFP-TDP-43^WT^ or GFP-TDP-43^G376D^ proteins and with control RNA or M10 siRNA. Seventy-two hours after transfection cells were lysed and cytoplasmic and nuclear fractions were separated. Samples were analysed by Western blotting using anti-TDP-43, anti-GAPDH, anti-histone H3 antibodies. Stain-Free Imaging Technology (Bio-Rad) was used for loading control. GAPDH and histone H3 were used. See Supplementary Figure 3 for uncropped blots. (**D**) Quantification of endogenous TDP-43 was shown. Data represent the mean ± SEM of at least three experiments. Statistical analysis was performed using one-way ANOVA followed by Tukey’s multiple comparisons test. *P < 0.05; ** < 0.01; ***P < 0.001; *F* = 4.945 (for nucleus); *F* = 3.710 (for cytoplasm).

To further confirm that the TDP-43^G376D^ mutation is responsible for cytoplasmic inclusion and that M10 can prevent TDP-43 aggregates, we transfected Neuro2A cells with a plasmid encoding GFP-TDP-43 wild-type or G376D and we treated them with control RNA or M10. Transfection with GFP-TDP-43^G376D^ led to the appearance of large cytoplasmic inclusions, which were no longer visible following M10 treatment, confirming that only the mutant form of TDP-43 forms aggregates and that this cellular phenotype is reverted following the treatment with allele-specific siRNA (Fig. 5A and B**).**

In cellular models, truncated forms of TDP-43 induced the mislocalization of endogenous TDP-43, which showed an altered nucleocytoplasmic distribution with the depletion of wild-type TDP-43 from the nucleus.^33,34^

In order to establish if mutant allele-specific silencing results in decreased retention of endogenous TDP-43 in the cytoplasm, we transfected HEK-293T with a plasmid coding for TDP-43 wild-type or the G376D mutant and with control RNA or M10. After 72 h, we lysed cells and separated nuclear and cytoplasmic fractions. Samples were subjected to Western blot analysis to evaluate the localization of endogenous TDP-43. The expression of TDP-43^G376D^ decreased the amount of endogenous TDP-43 in the nucleus and slightly increased its presence in the cytoplasm. Following M10 treatment, TDP-43 localization is similar to control (Fig. 5C and D). This result suggests that M10 treatment could enhance the amount of functional TDP-43 in the nucleus.

### Allele-specific siRNA ameliorated cell viability and oxidative stress in Neuro2A cells and ALS fibroblasts

**Fig. 6:**
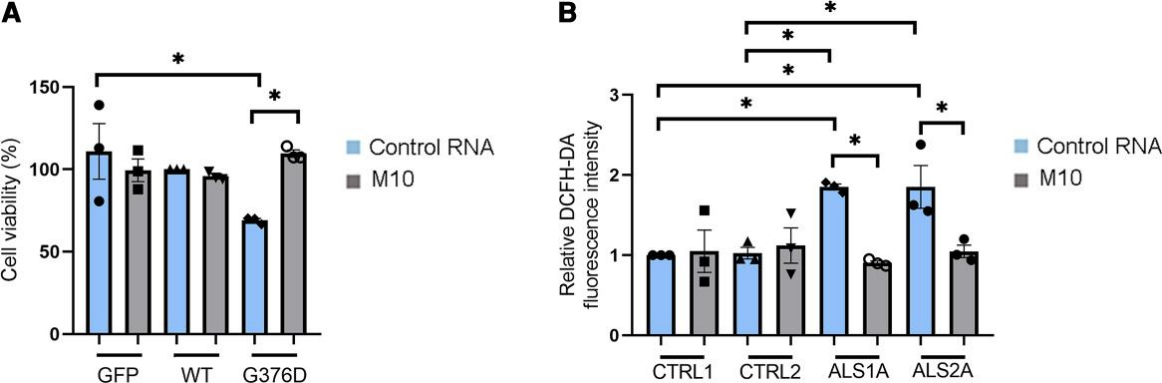
**M10 treatment rescues cell viability and oxidative stress.** (**A**) Neuro2A cells were transfected with plasmids encoding the GFP-TDP-43^WT^ or GFP-TDP-43^G376D^ proteins and with control RNA or M10 siRNA. Seventy-two hours after transfection, cells were subjected to SRB assay to measure cell viability. Data represent the mean ± SEM of at least three experiments. Statistical analysis was performed using one-way ANOVA followed by Tukey’s multiple comparisons test. *P < 0.05; ** < 0.01; ***P < 0.001; *F* = 4.060. (**B**) Fibroblasts isolated from two controls and two ALS patients carrying the TDP-43^G376D^ mutation (ALS1A, ALS2A) were transfected with control RNA and M10 for 6 days, treated with 10 µM DCFH-DA for 30 min and then lysed. Fluorescence intensity was read at 490 nm in a microplate reader. Data represent the mean ± SEM of at least three experiments. Statistical analysis was performed using one-way ANOVA followed by Tukey’s multiple comparisons test. *P < 0.05; ** < 0.01; ***P < 0.001; *F* = 5.946.

Having demonstrated that M10 treatment could reduce TDP-43 aggregation in the cytoplasm, we wondered if the treatment with this siRNA could help to improve cell viability and reduce oxidative stress in cellular models. Fang and co-workers^35^ demonstrated that inclusion bodies formed by full-length and truncated TDP-43 were neurotoxic. In order to evaluate if the expression of mutant TDP-43 affected cell viability, we performed an SRB assay. This method is based on SRB, which binds stoichiometrically to proteins under mild acidic conditions and can be extracted using basic conditions, thus, the bound dye reflects the number of cells.^36^ This assay demonstrated that expression of TDP-43^G376D^ reduced cell viability of Neuro2A cells (*P* = 0.0189). Importantly, M10 treatment restored cell viability to levels comparable to control cells (*P* = 0.0234; Fig. 6A).

Oxidative stress is elevated in ALS. Indeed, fibroblasts carrying the TDP-43^A382T^ and TDP-43^G294V^ mutations showed enhanced oxidative stress compared with control fibroblasts.^37^ We wondered if ALS1A and ALS2A patients showed this alteration and, to answer this question, we performed the DCFH-DA staining. DCFH-DA is taken up by cells where the acetyl groups were cleaved off by cellular esterase, resulting in DCFH. Oxidation of DCFH by ROS converts the molecule to dichlorofluorescein, which emits green fluorescence.^38^ Therefore, fluorescence intensity depends on the amount of ROS in cells. ALS1A and ALS2A patients showed increased oxidative stress compared with control cells (CTRL1/SLA1A *P* = 0.0268; CTRL1/SLA2A *P* = 0.0266; CTRL2/SLA1A *P* = 0.0336; CTRL2/SLA2A *P* = 0.0333). Notably, treatment of ALS fibroblasts with M10 reduced DCFH-DA fluorescence indicating an improvement of oxidative stress (ALS1A *P* = 0.0119; ALS2A *P* = 0.0403; Fig. 6B).

Altogether, these data suggest that the TDP-43^G376D^ mutation reduces cell viability and increases oxidative stress in cellular models and demonstrate that M10 treatment reverts both of these phenotypes.

## Discussion

In 1869, Jean–Martin Charcot defined ALS as a pure motor neuron disease, but now it is known that it is a multisystem neurodegenerative disorder characterized by clinical, genetic and neuropathological heterogeneity.^39^ Considering the genetic heterogeneity, ALS is associated with alterations in >20 genes. The most common genes involved are Chromosome 9 open reading frame 72 (*C9orf72*), SOD1, TDP-43, fused in sarcoma and TANK-binding kinase 1, which together cause 15% of the ALS cases.^40–42^

Here, we analysed an Apulian family from Lecce affected by familial ALS caused by the c. 1127G>A pathogenic variant in the *TARDBP* gene, which determines the aminoacidic substitution G376D in the TDP-43 protein and that it is inherited in autosomal dominant manner.^23^ This mutation has also been found in an Asian and a Swiss family with an autosomal dominant transmission and a clinical phenotype similar to that of the Italian family.^43,44^ Noteworthy, the Swiss family is genetically connected to the Apulian family (Association 2HE, personal communication).

Nowadays, ALS does not have an effective treatment. Indeed, the complexity of this disease has been further demonstrated by the failure of many randomized controlled trials, which did not lead to an improvement in disease progression or survival.^45^ Currently, the antiglutaminergic Riluzole is the most used drug in ALS patients, but the mean patient survival is prolonged by only 3–6 months.^46–48^ Hence, the need for new therapeutic strategies. One possibility is represented by antisense oligonucleotides (ASOs), short, synthetic, single-stranded oligodeoxynucleotides that bind a target mRNA, bringing it to degradation and reducing the levels of a toxic protein. Recently, this strategy received the approval of the Food and Drug Administration for Duchenne muscular dystrophy and spinal muscular atrophy, and several clinical trials are in progress.^49^ ASOs have also been considered for ALS.^50^ In the light of this, we designed allele-specific siRNAs for the c.1127G>A (p.G376D) mutation in TDP-43 and tested their efficacy in HEK-293T cells transfected with GFP-TDP-43 wild-type or G376D. We demonstrated that among the four ASOs designed, M10 reduced GFP-TDP-43^G376D^ mRNA and protein without affecting the wild-type (Fig. 2A–C).

Then, we tested M10 in fibroblasts derived from patients carrying the TDP-43^G376D^ mutation and belonging to the Italian family previously described.^23^ Also, in these cells, M10 effectively reduced the level of the mutated protein and mRNA (Fig. 3A–D). In particular, using primer pairs to discriminate against the wild-type and the mutated alleles, we demonstrated that only the G376D allele is affected by M10 treatment (Fig. 3C and D).

In 95% of ALS cases, the neuropathological signature of the disease is represented by the appearance of TDP-43 aggregates in the cytoplasm.^51^ In a previous work, TDP-43 was found in the cytoplasm of primary fibroblasts carrying *TARDBP* c*4G>A and p.A382T mutations.^52^ To test whether G376D fibroblasts are also characterized by this phenotype, we performed an immunofluorescence assay using an antibody able to recognize the C-terminal region of the TDP-43 protein. We demonstrated not only that ALS1A and ALS2A fibroblasts are rich in TDP-43 aggregates, which, on the contrary, were not present in control cells, but also that cells taken from Patient 1 at the onset of the disease were similar to controls. In contrast, those taken from the same patient at an advanced stage of the disease showed a cytoplasm rich in aggregates (Fig. 4A and B). This result highlighted that the formation of the aggregates takes place gradually and this phenomenon is associated with the progression of the disease, confirming that the loss of functional TDP-43 in the nucleus and its increased deposition in the cytoplasm is mostly seen in the end stages of the disease.^53^

We wondered if M10 could revert this phenotype; therefore, we treated fibroblasts with control RNA and M10 and we observed a reduction in the number of ALS cells showing aggregates following M10 treatment, while the cytoplasmic TDP-43 deposits were still visible in the cells of the same patient treated with control RNA (Fig. 4C and D).

Finally, to confirm that the aggregation of TDP-43 is specifically caused by the p.G376D mutation of TDP-43, we transfected Neuro2a cells with GFP-TDP-43 wild-type or mutant, demonstrating the appearance of cytoplasmic aggregates only in cells transfected with the plasmid encoding GFP-TDP-43^G376D^. Moreover, the treatment of these cells with M10 led to the disappearance of aggregates in this cellular model (Fig. 5A and B). Compared with fibroblasts from patients, in Neuro2a cells expressing the mutant protein, we observed the presence of single aggregates. This could possibly be related to the transient transfection method that we used. Indeed, several studies using this method reported cells with single aggregates.^54,55^ Larger aggregates are likely to accumulate in the cytoplasm with time.

Increasing evidence suggests that TDP-43 mislocalization determines cellular toxicity in ALS. This could be due to TDP-43 nuclear depletion, which alters several cellular processes.^12^ Through nuclear cytoplasmic separation, we evaluated the localization of endogenous TDP-43 and we demonstrated that the expression of p.G376D TDP-43 reduced the amount of endogenous TDP-43 in the nucleus, while M10 treatment, in TDP-43^G376D^-overexpressing cells increased nuclear TDP-43, possibly increasing functional protein levels (Fig. 5C and D).

To test whether the presence of mutated TDP-43 is associated with other phenotypic alterations, we evaluated the viability of Neuro2a overexpressing GFP, GFP-TDP-43 wild-type or GFP-TDP-43^G376D^ and treated the cells with control RNA or M10. Notably, the expression of mutated TDP-43 affected cell viability which was restored following M10 treatment (Fig. 6A). This allele-specific siRNA is also able to reduce the oxidative stress that characterizes ALS fibroblasts (Fig. 6B). Interestingly, recent work showed that mouse and human neurons are more sensitive to oxidative stress when TDP-43 aggregates are present since they sequester microRNAs and proteins, interfering with their function. Many of these proteins are mitochondrial proteins encoded by the nuclear genome and their alterations result in mitochondrial dysfunctions that increase oxidative stress. This gives rise to a vicious circle that could underlie ALS onset and progression.^56^ Considering our data, allele-specific silencing could put an end to this circle by reducing TDP-43 aggregation and oxidative stress and, maybe, ameliorating mitochondrial function. This point should be investigated in the future.

Further studies are necessary to confirm the efficacy of M10 in the treatment of ALS caused by the p.G376D mutation. An important point that should be considered is the effect of decreased TDP-43 levels caused by mutated allele silencing, considering that the overexpression but also the downregulation of TDP-43 may lead to neuronal dysfunction.^57^ However, in heterozygous null *TARDPB* mouse models the levels of TDP-43 are normal, suggesting tightly controlled compensation mechanisms following the loss of one allele.^58,59^ Indeed, TDP-43 can regulate the level of its own transcript *in vivo*.^60^ Another important point to consider regards the fact that TDP-43 aggregation mimics its knockdown since the expression of several proteins is affected in both conditions in the same way.^61^ Therefore, on the one hand, allele-specific silencing could reduce TDP-43 aggregation by restoring the functionality of several proteins, on the other hand, TDP-43 could regulate its own expression to compensate for the loss of the mutated allele. Clearly, this aspect should be evaluated further *in vivo*.

Still, our results are promising, considering the reversion of the phenotypes observed in the cellular models that we used. The results of Phase 1–2 trials based on the use of tofersen, an antisense nucleotide for ALS caused by SOD1 mutations, have been recently published. The treatment has proved to reduce SOD1 levels in the cerebrospinal fluid, but the study was not designed to test the safety and clinical efficacy of the drug.^62^ However, in October 2021, the results of another study were released. It was a 6-month Phase 3 randomized study, VALOR, which did not reach the primary endpoint represented by change in the ALS Functional Rating Scale-Revised (ALSFRS-R), even though signs of reduced disease progression were observed. Still, recently, at the European Network to cure ALS, Biogen Inc. presented new 12-month data that highlighted how starting tofersen treatment early slowed ALS progression. Indeed, the results showed a slowed decline of respiratory and clinical functions (measured by ALSFRS-R), strength and quality of life as well as a reduction in neurofilament, a marker of neurodegeneration (investors.biogen.com). Moreover, tofersen is being studied in the ATLAS study designed to evaluate the impact of initiating the treatment in the pre-symptomatic carriers of SOD1 variants.^63^

A further point that should be considered regards the application of siRNA treatment to neuronal diseases, which has limitations due to the lack of an efficient method of siRNA delivery in neurons. However, in recent years, several attempts have been made to chemically modify siRNAs in order to make them more effective.^64^ For instance, the generation of nanocomplexes or hydrophobically modified siRNAs were able to cause gene silencing in neurons *in vitro* and in mice.^65,66^

In the light of all this, the efficacy of M10 should be evaluated in neurons considering the possibility of chemically modifying it to improve its uptake by neurons and, in the future, it could be considered as a therapeutic agent in trials possibly evaluating its effects in the early stages of the disease.

## Conclusions

We identified a siRNA able to specifically silence the mutated allele of TDP-43. The treatment of TDP-43^G376D^ expressing cells with this siRNA strongly reduced the presence of TDP-43 aggregates in the cytoplasm. This treatment eased the burden of aggregates and thereby increased cell viability and diminished oxidative stress. This represents a potentially powerful therapeutic tool for the future that should be tested on iPS-derived neurons.

## Supplementary Material

fcac315_Supplementary_Data

## Data Availability

All data are available from the first author on request.

## Abbreviations

ALS: amyotrophic lateral sclerosis
ASOs: antisense oligonucleotides
DCFH-DA: 2′,7′-dichlorodihydrofluorescein diacetate
GAPDH: glyceraldehyde-3-phosphate dehydrogenase
GFP: green fluorescent protein
ROS: reactive oxygen species
RRM: RNA recognition motifs
SDS: sodium dodecyl sulphate
SEM: standard error mean
siRNA: small interfering RNA
SOD1: superoxide dismutase 1
TDP-43: TAR DNA-binding protein 43
