# Supplementary material for: Allele-specific silencing as therapy for familial amyotrophic lateral sclerosis caused by the p.G376D TARDBP mutation
Source: Brain Commun. 2022 Dec 16;4(6):fcac315. doi: 10.1093/braincomms/fcac315 (PMC9897181; doi:10.1093/braincomms/fcac315)
Supplement: fcac315_Supplementary_Data [file fcac315_Supplementary_Data.pdf]

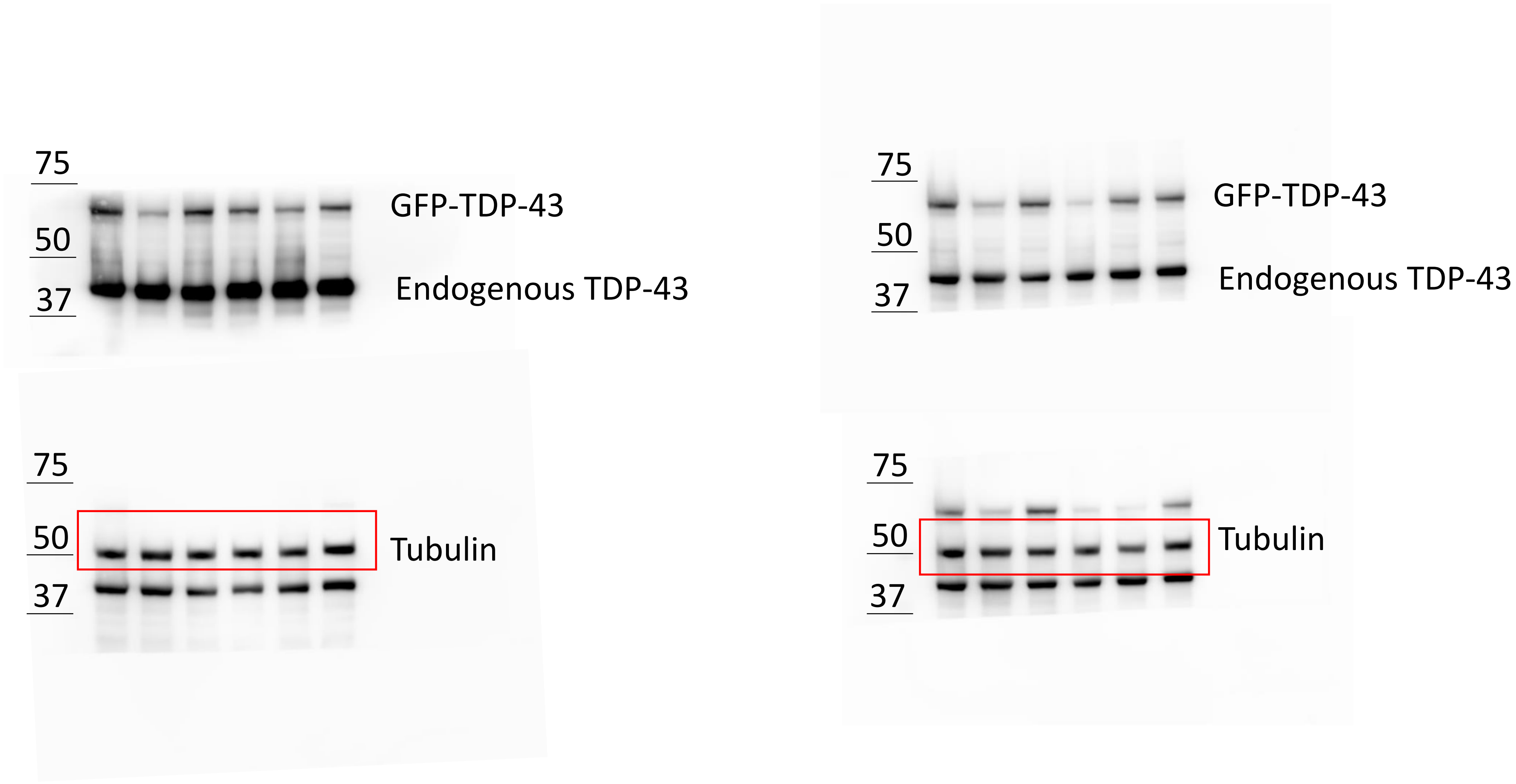

Supplementary Figure 1. Uncropped blots for figure 2A

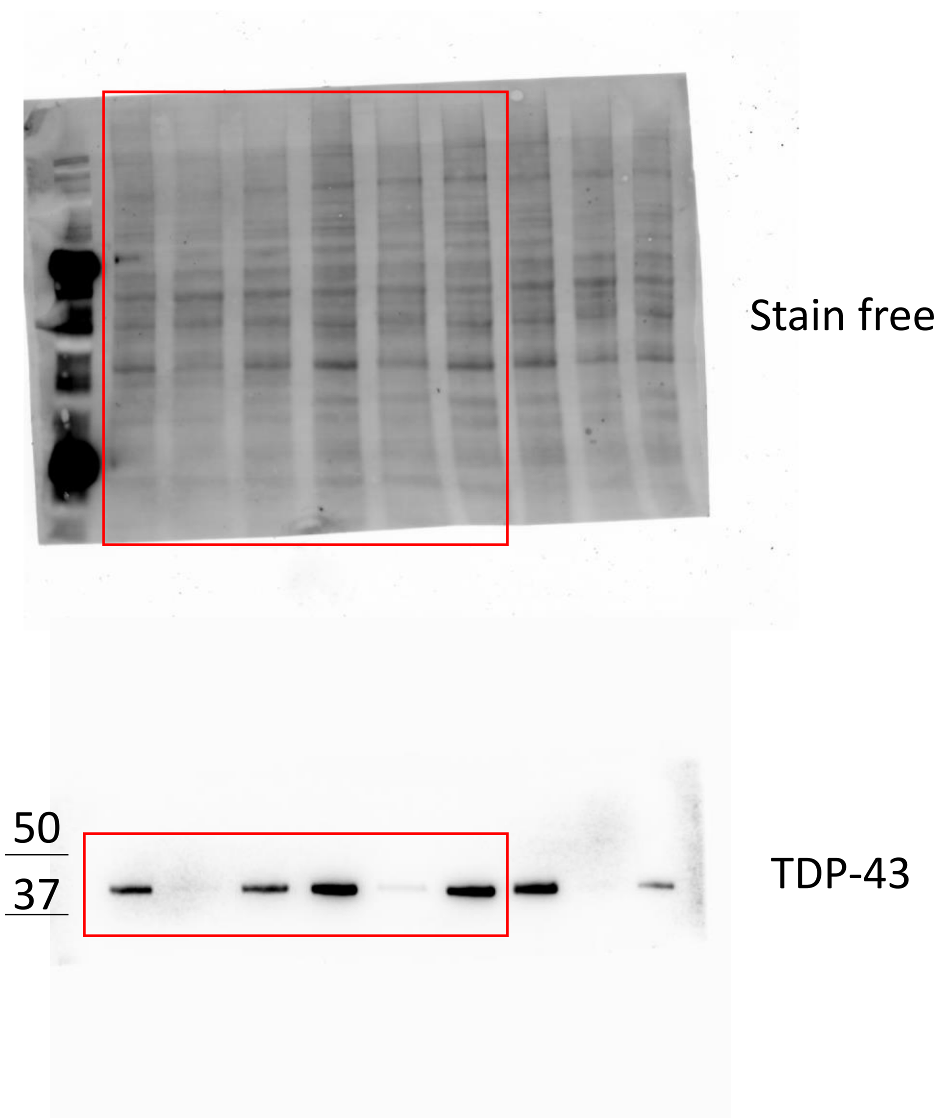

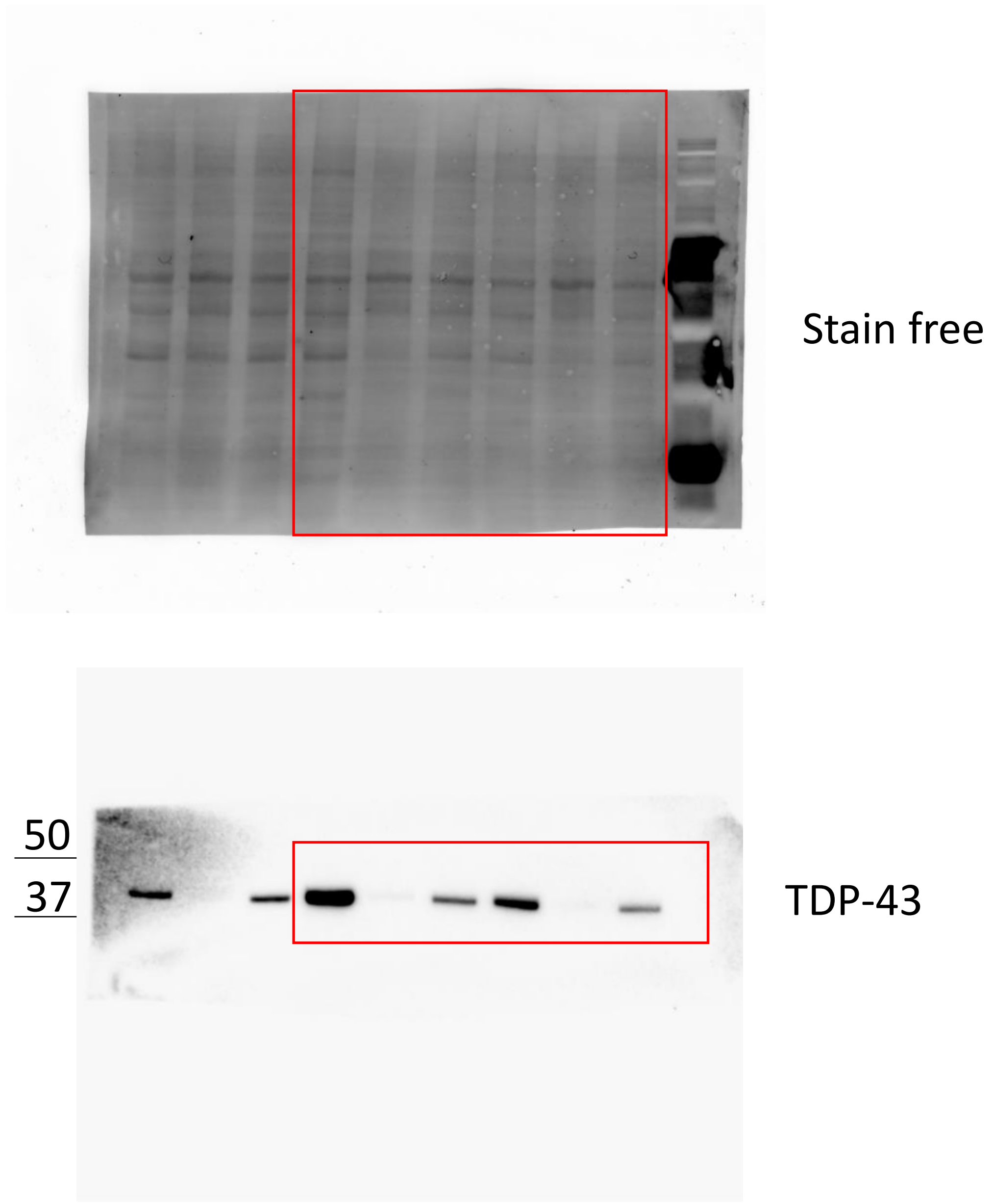

Supplementary Figure 2. Uncropped blots for figure 3A

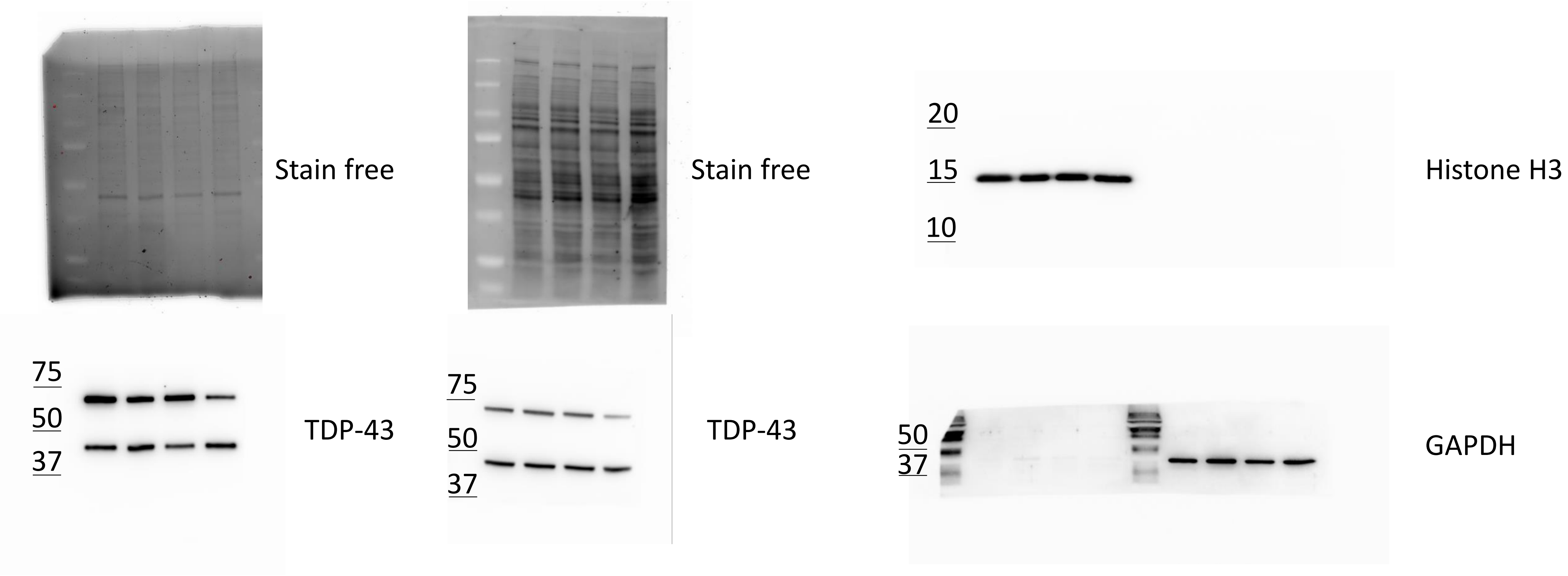

Supplementary figure 3. Uncropped blots for figure 5C
